# Performance of Osteoporosis Self-Assessment Tool (OST) in Predicting Osteoporosis—A Review

**DOI:** 10.3390/ijerph15071445

**Published:** 2018-07-09

**Authors:** Shaanthana Subramaniam, Soelaiman Ima-Nirwana, Kok-Yong Chin

**Affiliations:** Department of Pharmacology, Faculty of Medicine, Universiti Kebangsaan Malaysia, Jalan Yaacob Latif, Bandar Tun Razak, Cheras, Kuala Lumpur 56000, Malaysia; shaanthana_bks@hotmail.com (S.S.); imasoel@ppukm.ukm.edu.my (S.I.-N.)

**Keywords:** bone mineral density, dual-energy X-ray absorptiometry, mass screening, osteopenia, sensitivity, specificity

## Abstract

Bone health screening plays a vital role in the early diagnosis and treatment of osteoporosis to prevent fragility fractures among the elderly and high-risk individuals. Dual-energy X-ray absorptiometry (DXA), which detects bone mineral density, is the gold standard in diagnosing osteoporosis but is not suitable for screening. Therefore, many screening tools have been developed to identify individuals at risk for osteoporosis and prioritize them for DXA scanning. The Osteoporosis Self-assessment Tool (OST) is among the first tools established to predict osteoporosis in postmenopausal women. It can identify the population at risk for osteoporosis, but its performance varies according to ethnicity, gender, and age. Thus, these factors should be considered to ensure the optimal use of OST worldwide. Overall, OST is a simple and economical screening tool to predict osteoporosis and it can help to optimize the use of DXA.

## 1. Introduction

Osteoporosis is a progressive bone metabolic disease. It is undetectable until a bone fracture occurs. Once osteoporosis has developed, then it is less likely to completely restore the bone strength of the patients [1]. The prevalence of osteoporosis is increasing as the global population ages rapidly. In Asia, the number of osteoporotic hip fractures is expected to rise from 1,124,060 in 2018 to 2,563,488 in 2050 [2]. The Asians also encompassed 55% of the population at risk for fragility fractures worldwide [3]. The escalating morbidity and mortality rates due to osteoporotic fractures have distressed the patients, families, and society [4]. In addition, fragility fractures also contribute to a tremendous healthcare and economic burden. A recent meta-analysis of studies in Asia indicated that the median medical cost for hip fracture was USD 2943, representing around 19% of the gross domestic product of the countries studied in 2014 [5]. Thus, it is crucial to identify individuals at risk for osteoporosis to enable early intervention for fracture prevention.

Dual-energy X-ray absorptiometry (DXA) is the gold standard technique used to detect osteoporosis. According to the World Health Organization (WHO), a bone mineral density (BMD) ≤ −2.5 standard deviations (SD) below the young adult mean (or a T-score ≤ −2.5) indicates osteoporosis, while a T-score value at any site between ≤−1.0 and >−2.5 indicates a low bone mass or osteopenia [6]. Dual-energy X-ray absorptiometry cannot be widely used for osteoporosis screening due to its high cost and limited availability in most developing countries [7]. Quantitative ultrasound (QUS) has been developed as an alternative to DXA for osteoporosis screening [7]. Although QUS is portable and more economical than DXA, it may be unavailable in all primary medical settings.

Various clinical risk assessment tools have been developed to evaluate the risk of osteoporosis [8]. These screening tools help physicians to prioritize high-risk patients for a DXA scan. Some of the screening algorithms are the Fracture Risk Assessment Tool (FRAX), Qfracture algorithm, and Garvan Fracture Risk Calculator (Garvan) [9]. The Osteoporosis Self-assessment Tool (OST) is another predictive algorithm currently in use to predict the risk for osteoporosis [10]. It was first established by Koh et al. (2001) using data of postmenopausal women from eight Asian countries. The screening algorithm was only based on age (years) and body weight (kg): OSTA score = (body weight − age) × 0.2, with three osteoporosis risk categories: low risk (>−1), moderate risk (−1 to −4), and high risk (<−4). It performed well to determine women at risk of osteoporosis [11]. The performance of OST among Asian men was first assessed by Kung et al. (2004) and it demonstrated a moderate performance in predicting osteoporosis [12]. OST has been known as OSTA (OST for Asians) when it is applied to Asian women. The establishment of OSTA only involved postmenopausal women and men from East and Southeast Asia. A recent article by Chin (2017) reviewed the performance of OSTA among various Asian populations, but the performance of OST in non-Asian countries was not examined [13]. Thus, the present review summarized and compared the performance of OST in determining osteoporosis risk among the Asian and non-Asian population.

## 2. Literature Search

**Figure 1:**
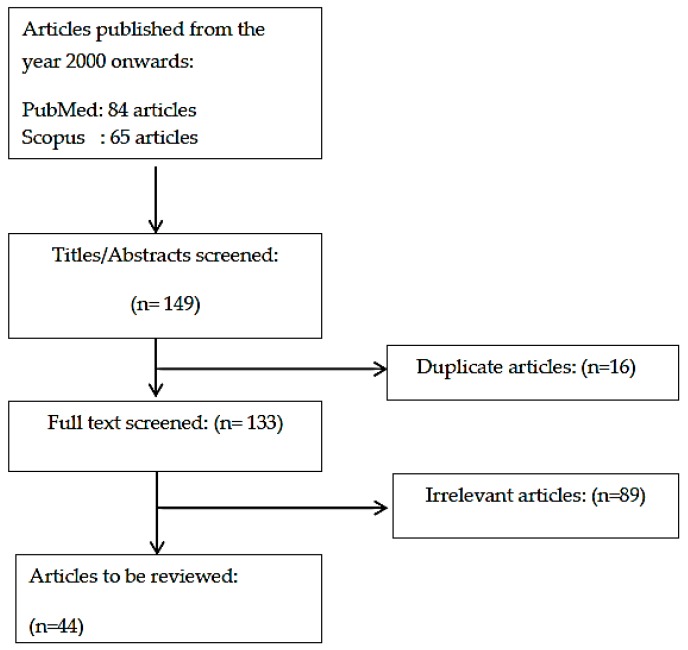
Flowchart of literature search.

A literature search was performed from 15 January 2018 to 4 April 2018 using two databases: PubMed and Scopus. Only original articles written in English were included in this review. The search term used was “*osteoporosis self-assessment tool*”. The search revealed 84 articles from PubMed and 65 articles from Scopus, which resulted in a total of 149 articles. After removing 16 duplicated articles, 133 articles were screened based on title and abstract. Only studies investigating the performance of OST against DXA were considered. The present review included 44 relevant articles (Figure 1).

## 3. Performance of OST among Asians

### 3.1. Performance of OST among Asian Women

The osteoporosis self-assessment tool for Asians (OSTA) was first developed by Koh et al. (2001) using data of postmenopausal women from eight Asian countries. The final algorithm only selected age and body weight as the predictors, creating the formula: OSTA score = (body weight in kg − age in years) × 0.2. Based on the truncated product of this formula, the women could be divided into three risk categories: low-risk (>−1), moderate-risk (−1 to −4), and high-risk (<−4). The predictive values of these scores were good, as indicated by the fact that 61% women categorized as high-risk were osteoporotic compared to 3% in the low-risk group in their study [11]. Its performance (cutoff = −1; sensitivity = 91%; specificity = 45%; AUC = 0.79) was superior than the reported values of the SOFSURF index [14], Osteoporosis Risk Assessment Index (ORAI), and Simple Calculated Osteoporosis Risk Estimation (SCORE) [15]. The performance of OSTA was subsequently validated in other East Asian populations, such as Chinese [16] and Korean women [17], and comparable results were obtained. This is not surprising, considering that a majority of the subjects in the development phase were East Asians [11]. In addition, the validation study by Huang et al. (2015) showed that OSTA performed better when BMD at the femoral neck was used as the reference, and when the women tested were older [16]. The site difference is probably due to the presence of osteophyte at the lumbar spine, which distorts its BMD. This finding was validated in similar studies conducted among Thai women [18,19]. The age difference coincides with the development population, which was elderly women (mean age: 62.3 ± 6.2 years) [11]. The limitation of OSTA in predicting osteoporosis among younger women was also observed in Thai women [19].

### 3.2. Performance of OST among Asian Men

Men also suffer from osteoporosis and their post-fracture mortality rate is higher than women [20]. Therefore, OSTA was developed for men by Kung et al. in 2004 based on data of community-dwelling Chinese men (age range: 50–93 years) in Hong Kong. The algorithm and cutoff values were the same as reported by Koh et al. (2001), but its performance in the development and validation cohort was not as good as in postmenopausal women (cutoff = −1; sensitivity = 71–73%, specificity = 68%; AUC = 0.780–0.790) [12]. OSTA was subsequently validated in the Chinese Han population [21] and Korean men [22], and both studies obtained a sensitivity > 80%. In contrast, OSTA (cutoff < −1) demonstrated a low sensitivity (27.6–28.5%) and a high specificity (89.2–92.7%) in a large study involving Chinese men of a wide age range (40–96 years) [23]. Sub-analysis revealed that similar to women, OSTA only performed well among older subjects [23].

### 3.3. Performance of OST with Modified Cutoff Values

Since the original cutoff values for OSTA were established in postmenopausal elderly women, predominantly Eastern Asians, its performance, in terms of sensitivity and specificity, may vary according to sex, age, and ethnic groups. Hence, modification to the cutoff values may be necessary to ensure the optimal performance of OSTA. This hypothesis was tested by Bhat et al. (2016) among Indian subjects (aged > 50 years) using a cutoff of 2 and OSTA achieved high sensitivity (sensitivity = 95.7%, specificity = 33.6%, AUC = 0.702) in predicting osteoporosis among the subjects [24]. However, OSTA failed to show similar performance in other populations (Chinese men with the cutoff −3.5, sensitivity = 47.3% [4]; Taiwanese men with the cutoff −1.86, sensitivity 69.2% [25]). In the subsequent discussion, the readers should notice the changes in cutoff values in many studies.

### 3.4. Performance of OST in Comparison with Other Screening Tools

QUS is another popular osteoporosis screening tool [7]. It was reported to have a strong association with BMD and bone mineral content (BMC) measured by DXA [26]. In three studies, OSTA was found to perform equally with QUS [8,27,28]. Thus, OSTA can be used in medical settings without QUS. However, it should be noted that OSTA and QUS cannot be used interchangeably because the two tools are not equivalent [29].

OSTA was also compared against other screening algorithms. With a modified cutoff of −2, OSTA showed a sensitivity of 90.0–91.9% among postmenopausal women, which was better than SCORE (cutoff ≥ 8), ORAI (cutoff ≥ 20), ABONE (cutoff = 3), and WEIGHT (cutoff < 54 kg) [30]. In another study among the Taiwanese elderly, OSTA achieved a sensitivity of 100% in men and women, outperforming ABONE (cutoff ≥ 2), BWC (<70 kg), FRAX and GARVAN (cutoff > 3% for hip fracture, > 20% for major osteoporotic fracture), ORAI (cutoff ≥ 9), OSIRIS (cutoff ≤ 1), OSTA (cutoff < −1), and SCORE (cutoff ≥ 6) [27].

The universality of OSTA was challenged by some researchers with osteoporosis screening algorithms designed for the local populations. The performance of OSTA was proven to be equivalent in some cases. For example, the Khon Kaen Osteoporosis Study (KKOS) (cutoff < −1) scoring system shared a similar performance with OSTA (cutoff < −1) in Thai women (AUC: 0.64 vs. 0.65) [31]. The Beijing Friendship Hospital Osteoporosis Self-Assessment Tool (BFH-OST) also performed similarly to OSTA (cutoff = −1) in predicting osteoporosis in Chinese Han women and men (AUC: 0.795–0.797 vs. 0.732–0.782), despite having a higher sensitivity (73.58–89.92% vs. 50.42–65.28%) [32,33]. Among the South Indian elderly, the performance of the Male Osteoporosis Risk Estimation Score (MORES) (cutoff = 6) was also equivalent to OSTA (cutoff ≤ 2) (AUC: 0.760 vs. 0.778) [34].

In some cases, the local algorithms were better than OSTA in osteoporosis screening. Among Taiwanese women, the Osteoporosis Preclinical Assessment Tool (OPAT) containing four predictors (age, menopausal status, weight, and alkaline phosphatase activity) performed better than OSTA (sensitivity: 87% vs. 78%; AUC: 0.77 vs. 0.69) [35]. Similarly, the Korean Osteoporosis Risk-Assessment Model (KORAM) (cutoff < −9) also performed better than OSTA in predicting osteoporosis among Korean menopausal women (cutoff < 0) (AUC: 0.682–0.709 vs. 0.617–0.626) [36]. The Malaysian Osteoporosis Screening Tool (MOST) (cutoff ≥ 4) was also superior to OSTA (cutoff < 2) (AUC: 67.6% vs. 52%) in predicting osteoporosis among healthy women [37]. Despite that, these local screening tools might not be useful outside the local settings.

A summary of the literature on the performance of OST among Asians is listed in Table 1.

## 4. Performance of OST among Non-Asians

### 4.1. Performance of OST among Non-Asian Women

Although developed for Asians, the performance of OST has also been validated in non-Asians. The algorithm is the same as reported by Koh et al. (2001), but the cutoff values have been optimized to suit the designated populations. The performance of OST (cutoff < 2) was good in determining Caucasian women at risk of osteoporosis (sensitivity = 86–95.3%; specificity = 39.6–40%; AUC = 0.726–0.82) [10,38]. A study indicated that the performance of OST was similar between younger (cutoff ≤ 1 for 45–64 years) and older women (and cutoff ≤ −1 for >65 years) when different cutoff values were used [39].

### 4.2. Performance of OST among Non-Asian Men

The use of OST in predicting bone health among non-Asian men was also validated. In Caucasian men, OST was able to predict individuals with osteoporosis (sensitivity = 93%, specificity = 66%, AUC = 0.836) [40]. The performance was better when BMD at total hip (sensitivity = 87.5%; specificity = 58.2%; AUC = 0.787) was used as the reference compared to the lumbar spine (sensitivity = 63.6%; specificity = 59.5%; AUC = 0.66) [41]. When operated in younger men (≥35 years), OST performed optimally when the cutoff was modified to <4 (sensitivity = 83%, specificity = 57%, AUC = 0.83) [42].

### 4.3. Performance of OST with Modified Cutoff Values

The cutoff values of OST need to be optimized in different non-Asian populations. In comparison to the original −1 for Asians, a cutoff ≤ 2 was tested for Caucasian to obtain a similar sensitivity value [43]. Other cutoff values, such as < 3 for Portuguese men (sensitivity = 75.5%, specificity = 50.0%, AUC = 0.632) [44], < 6 for veteran US men (sensitivity = 82.6%, specificity = 33.6%, AUC = 0.67) [45], and < 4 for African men (sensitivity = 83%, specificity = 57%, AUC 0.83) [42], have been adopted previously. The non-Asians’ cutoff values are generally higher than Asians’ because they have a higher body weight.

Even within the same ethnicity, the cutoff values of OST need to be modified based on sex. A Spanish study showed that OST performed optimally at a cutoff of 2 in women (sensitivity = 94%, specificity = 59%, AUC = 0.762) and 3 in men (sensitivity = 39%, specificity = 86%, AUC = 0.623) [46].

In a group of men with rheumatoid arthritis, OST was weak (sensitivity = 64%; specificity = 54%) in determining those with low BMD even though the cutoff was modified to <4. The researchers indicated that the performance of OST could be limited by the low lean body mass of patients with rheumatoid arthritis [47].

### 4.4. Performance of OST in Comparison with Other Screening Tools

In comparison with other screening algorithms, OSTA (at various optimized cutoff values) performed similarly to ABONE [48], SCORE [10,48,49,50,51,52,53], SOFSURF [49,51], ORAI [10,48,49,51,52], OSIRIS [10,49], United States Preventive Services Task Force (USPSTF)–FRAX [52,53,54], RF [52], BMI [52], pBW [49], Weight Criterion [48], and QUS [49] in women. It also performed similarly to the male-specific screening tool, Mscore, developed by Zimering et al. (2007) [55]. Only the study of Hawker et al. (2012) reported that OST was weak (sensitivity = 47%, AUC = 0.69) in identifying women with low BMD when compared to a new screening tool developed by the study (sensitivity = 93%, AUC = 0.75) [56].

A summary of the literature on the performance of OST among non-Asians is listed in Table 2.

## 5. OST for Fracture Prediction

Fragility fracture is one of the most common complications of osteoporosis. Although OST was developed to identify individuals with low BMD, its ability to predict fracture risk was assessed in several studies [54,58,59]. Yang et al. (2013) reported that OSTA (cutoff < −1) performed well in determining new vertebral fractures among postmenopausal Chinese women (sensitivity = 81.7%; specificity = 66%; AUC = 0.812) [59].

In comparison with other fracture prediction tools, the performance of OST was weaker than DXA [58] and FRAX [58] in Chinese men and the Singh index in patients with type 2 diabetes mellitus [60]. However, a study showed that USPSTF (FRAX) (sensitivity = 25.8%; specificity = 83.3%; AUC = 0.56) was not better than OST in fracture prediction (sensitivity = 39.8%; specificity = 60.7%; AUC = 0.52) among non-Asian postmenopausal women [54].

A summary of the literature on the performance of OST to predict fracture risk is listed in Table 3.

## 6. Conclusions

The performance of OST in predicting osteoporosis has been tested in various Asian and non-Asian populations. It demonstrates good predictive values in terms of sensitivity, specificity, and AUC when BMD is used as the reference. Some modifications in the OST cutoff should be made and tested to optimize its performance prior to its deployment, since the performance may vary according to age, sex, ethnicities, and the site of BMD measurement. Validation studies are necessary before including OST in the national guideline for osteoporosis screening. In most studies, OST demonstrated a high sensitivity and low specificity, which is typical for a screening test. In other words, OST might direct some individuals with normal bone health for an unnecessary DXA scan. At the same time, the number of potential patients subjected to a DXA scan is maximized, allowing the early detection and treatment of osteoporosis. This will reduce the complications and burdens of the disease. Thus, we argue that the benefits of implementing OST will outweigh its cost. As a conclusion, OST is a useful osteoporosis screening tool in prioritizing high-risk individuals for a DXA scan. It enables early disease detection, optimizes the use of the diagnostic facility, and therefore reduces the disease burden of osteoporosis.

## Figures and Tables

**Table 1:** Performance of OST among Asians.

Study	Objective	Subject Description	Number of Subjects Recruited	Methods	Cutoff	Sensitivity (%)	Specificity (%)	AUC	Remarks
Koh et al. (2001) [11]	To develop Osteoporosis Screening Tool for Asians (OSTA)	Postmenopausal women (mean age 62 years) recruited from 21 clinics in eight Asia countries.	860	DXA: 8 Hologic machines (3 Model 4500, 5 Model 2000), 4 Norland (2 XR-36, 1 S-26, 1 XR-26), 12 Lunar (3 DPX-IQ. 6 DPX-L, 3 Expert) machines	OSTA < −1 T-score< −2.5	91	45	0.79	
					SOFSURF < 1.4 T-score < −2.5	90	46	0.77	
					ORAI < 15 T-score < −2.5	84	52	0.76	
					SCORE < 10 T-score < −2.5	90	33	0.77	
Park et al. (2003) [17]	To validate the effectiveness of OSTA in identifying osteoporosis among Korean women	Postmenopausal women from a clinic in Korea and who were not on hormone replacement therapy (mean age: 59.1 ± 7.7 years)	1101	DXA GE Lunar model DPQ-IQ BMD at FN	OSTA < −1 T-score ≤ −2.0	80	72	0.85	Single-centered
					OSTA < −1 T-score ≤ −2.5	87	67	0.873	
Geater et al. (2004) [18]	To validate the performance of OSTA in predicting osteoporosis among Korean women	Thai post-menopausal women (mean age: 60.5 ± 9.7 years) without risk of osteoporosis	388	DXA Lunar, Madison BMD at FN and LS	OSTA < −1 FN T-score < −2.5	93.5	60.8	Value not mentioned	
					OSTA < −1 LS T-score < −2.5	79.5	69.5	Value not mentioned	
					OSTA < 0 FN T-score < −2.5	93.5	29.8	Value not mentioned	
					OSTA < 0 LS T-score < −2.5	92.4	35.7	Value not mentioned	
Huang et al. (2015) [16]	To determine the performance of OSTA among middle-aged and old women	Healthy women (age range: 40–96 years) from a hospital in Chengdu region, China	15,752	DXA (Lunar Prodigy- GE Healthcare, Madison, WI, USA) BMD at LS, FN, and TH	OSTA < −1 LS T-score < −1	56.9	87.7	0.812	
					OSTA < −1 LS T-score < −2.5	77.3	73.5	0.812	
					OSTA < −1 FN T-score < −1	56.2	89.8	0.822	
					OSTA < −1 FN T-score < −2.5	88.1	69.3	0.822	
Yang et al. (2015) [21]	To validate OSTA among elderly males to determine the risk of primary osteoporosis	Healthy males (mean age: 65.17± 9.29 years)	245	DXA (Hologic, Inc., Bedford, MA, USA) BMD at LS and LF	OSTA < 1 FN T-score < −2.5	84	49	0.712	
					OSTA < 1 TH T-score < −2.5	Value not stated	Value not stated	0.658	
					OSTA < 1 LS T-score < −2.5	Value not stated	Value not stated	0.535	
Oh et al. (2016) [22]	To compare the effectiveness of Korean Osteoporosis Risk-Assessment Model for Men (KORAM-M) and OSTA	Men aged 50 and above from 2009 and 2010 Korean National Health and Nutrition Examination Survey	Development phase: 1340 Validation phase: 1110	DXA Hologic Discovery BMD at FN or LS	Development: OSTA < −1	90.8	36.9	0.639	
					KORAM-M < −9	90.8	42.4	0.666	
					Validation: OSTA < −1	92.3	33.2	0.627	
					KORAM-M < −9	87.9	39.7	0.638	
Huang et al. (2017) [23]	To assess the effectiveness of OSTA using various cutoffs	Healthy men aged 40–96 years recruited from a hospital in Chengdu region, China	11,039	DXA (GE Lunar, Madison, WI, USA) BMD at LS and FN	OSTA < −1 LS T-score < −1	27.6	89.2	value not stated	
					OSTA < −1 LS T-score ≤ −2.5	57.3	86.7		
					OSTA < −1 FN T-score < −1	28.5	92.7		
					OSTA < −1 FN T-score ≤ −2.5	65.9	87.0		
Bhat et al. (2017) [24]	To evaluate the performance of OSTA in predicting OP among Indian men	Indian men above 50 years and without apparent risk of OP	257	DXA (QDR 4500 A, Hologic Inc., Bedford, MA, USA) BMD at LS, TH and FN	OSTA ≤ 2 T-score at any sites ≤ −2.5	95.7	33.6	0.702	
Zha et al. (2014) [4]	To validate OSTA and QUS and their combination in predicting OP among the high-risk population	Chinese men (mean age: 78.0 years)	472	DXA (Discovery A, Hologic, USA) QUS (Sahara clinical bone sonometer- Hologic) BMD at LS and LH	OSTA < −3.5 FN T-score < −2.5	65.5	74.8	0.724	Small sample size Sample recruited from a single centre
					OSTA < −3.5 TH T-score < −2.5	81.8	72.7	0.787	
					OSTA < −3.5 LS T-score < −2.5	45.4	74.7	0.652	
					OSTA < −3.5 T-score at any site < −2.5	47.3	76.8	0.676	
					QUS < −1.15 FN T-score < −2.5	88.9	47.4	0.762	
					QUS < −2.15 TH T-score < −2.5	82.4	86.6	0.883	
					QUS < −1.25 LS T-score < −2.5	82.7	57.9	0.750	
					QUS < −1.25 T-score at any site < −2.5	80.4	59.7	0.762	
Chang & Yang (2016) [25]	To conduct a cutoff study among males by using OST, BMI, age and body weight	Retrospective data of Northern Taiwan males with mean age of 71.9 ± 13.3 years	834	DXA BMD at FN	OST < −1.86 T-score ≤ −2.5	69.2	63	0.70	Subjects were patients referred to BMD test by orthopaedic surgeons
					BMI < 23 kg/m^2^ T-score ≤ −2.5	60.4	61.6	0.63	
					Weight < 58.8 kg T-score ≤ −2.5	43.9	78.2	0.66	
Kung et al. (2003) [28]	To develop OSTA for Asian men	Community-dwelling Chinese men (age: 50–93 years)	420	Development followed by validation in 356 men DXA: QDR 2000 Plus Hologic, Waltham, MA, USA BMD at LS and LF QUS: Sahara Hologic, Waltham, MA, USA	Development: OSTA < −1 T-score ≤ −2.5	73	68	0.790	Subjects were not selected randomly
					Validation: OSTA < −1 T-score ≤ −2.5	71	68	0.780	
					Validation: QUI < −1.2 T-score ≤ −2.5	76	72	0.80	
					Either OSTA <−1 or QUI < −2.5 T-score ≤ −2.5	88	64	0.82	
Chan et al. (2006) [30]	To compare the validity of various OP risk indices in elderly Chinese females	Community-dwelling postmenopausal women (age ≥55)	135	DXA (Hologic QDR 4500 W) BMD at FN and LS	OSTA (cutoff ≤ −2 FN T-score ≤ −2.5 LS T-score ≤ −2.5	90.9	58.8	0.82	Small sample size
						91.9	42.9	0.73	
					SCORE (cutoff ≥ 8) FN T-score ≤ −2.5 LS T-score ≤ −2.5	93.9	60.8	0.80	
						86.5	60.2	0.72	
					ORAI (cutoff ≥ 20) FN T-score ≤ −2.5 LS T-score ≤ −2.5	75.8	66.7	0.76	
						62	62	0.68	
					ABONE (cutoff = 3) FN T-score ≤ −2.5 LS T-score ≤ −2.5	81.8	55.9	0.70	
						73	54.1	0.66	
					SCORE (cutoff ≥ 8) FN T-score ≤	67.9	77.5	0.78	
						62.2	76.5	0.73	
Chaovisitsaree et al. (2007) [19]	To compare OSTA with DXA in determining osteopenia and osteoporosis menopausal women	Thai menopausal women (age range: 45–87 years) from Menopause Clinic in Chiang Mai University	315	DXA BMD at FN, LS and radius	OSTA < −1 LS T-score ≤−1 FN T-score ≤−1 Radius T-score ≤−1	36.2	71.4	Value not mentioned	
						40.6	72.0		
						48.3	75.1		
					OSTA < −1 LS T-score ≤−2.5 FN T-score ≤−2.5 Radius T-score ≤−2.5	45.8	68.9		
						75.0	67.8		
						60	68.5		
Chen et al. (2016) [27]	To compare the performance of different screening tools to predict fracture or OP risk among older people	Community-dwelling older people aged 60 and above (mean age: 67.4 ± 6,4 years) recruited from Tanzi District, Taiwan	553	DXA Hologic Discovery Wi Bone Densitometer BMD at FN QUS GE Lunar, Madison, WI	QUS FN T-score ≤ −2.5	20 (M) 59 (F)	86 (M) 75 (F)	0.72(M) 0.77(F)	
					ABONE ≥ 2	100 (M) 100 (F)	28 (M) 10 (F)	0.78(M) 0.70(F)	
					BWC < 70 kg	100 (M) 100 (F)	36 (M) 7 (F)	0.92(M) 0.80(F)	
					FRAX Hip fracture (>3%)	80 (M) 83 (F)	71 (M) 54 (F)	0.86(M) 0.75(F)	
					MOF (>20%)	0 (M) 17 (F)	99 (M) 96 (F)	0.77(M) 0.71(F)	
					GARVAN Hip fracture (>3%)	60 (M) 28 (F)	79 (M) 95 (F)	0.72(M) 0.80(F)	
					Any osteoporotic fracture (>20%)	20 (M) 55 (F)	96 (M) 73 (F)	0.72(M) 0.75(F)	
					ORAI ≥ 9	100 (M) 100 (F)	19 (M) 5 (F)	0.87(M) 0.77(F)	
					OSIRIS ≤ 1	100 (M) 100 (F)	29 (M) 6 (F)	0.94(M) 0.83(F)	
					OSTA ≤ −1	100 (M) 100 (F)	58 (M) 27 (F)	0.94(M) 0.83(F)	
					SCORE ≥ 6	100 (M) 100 (F)	45 (M) 15 (F)	0.91(M) 0.80(F)	
Chen et al. (2017) [35]	To establish a prediction model to identify osteopenia risk in women aged 40–55 years	Taiwanese women recruited from a health checkup centre	1350	DXA (DPX-L; GE Lunar Health Care, Madison, WI, USA) BMD at LS	OSTA ≤ 1	78	47	0.69	Novel algorithm to predict osteopenia
					OPAT ≥ 1 −1 ≥ T-score > −2.5 at LS	87	42	0.77	
Panichyawat & Tanmahasamut (2012) [31]	To compare the performance of OSTA and Khon Kaen Osteoporosis Study (KKOS) scoring system to predict OP among postmenopausal women in Thailand	Postmenopausal women (mean age: 55.8 ± 5.9 years) from menopause clinic	441	DXA BMD at FN and TH	OSTA = −1 T-score at any site ≤ −2.5	51.7	77.4	0.65	Subjects from a single centre
					OSTA = 0 T-score at any site ≤ −2.5	66.7	57.1	0.62	
					KKOS = −1 T-score at any site ≤ −2.5	56.3	71.8	0.64	
					KKOS = 0 T-score at any site ≤ −2.5	57.5	67.2	0.62	
Oh et al. (2013) [36]	To develop Korean Osteoporosis Risk-Assessment Model (KORAM) and compare its performance with OSTA	Postmenopausal women who participated in the 2009 and 2010 Korean National Health and Nutrition Examination Survey	Development: 1209 Validation: 1046	DXA QDR Discovery, Hologic BMD at TF, FN and LS	Development: OSTA < 0 FN or LS T-score < −2.5	96.8	28.3	0.626	
					OSTA < 0 FN or LS T-score < −2.0	93.7	34.6	0.641	
					KORAM < −9 FN or LS T-score < −2.5	91.2	50.6	0.709	
					KORAM < −9 FN or LS T-score < −2.0	85.2	60.1	0.726	
					Validation: OSTA <0 FN or LS T-score < −2.5	94.2	29.2	0.617	
					OSTA < 0 FN or LS T-score < −2.0	90.9	35.0	0.629	
					KORAM < −9 FN or LS T-score < −2.5	84.8	51.6	0.682	
					KORAM < −9 FN or LS T-score < −2.0	79.2	60.2	0.697	
Lim et al. (2011) [37]	To develop and validate Malaysian Osteoporosis Screening Tool (MOST) to detect low BMD in Malaysia	Healthy women (mean age: 51.3 ± 5.4 years) from a residential area	Development: 514 Validation: 72	DXA Norland XR-36 BMD at FN and LS	OST < 2 FN T-score ≤−2.5	88	52	Value not mentioned	
					ORAI > 8 FN T-score ≤−2.5	90	52		
					SCORE > 7 FN T-score ≤−2.5	89	58		
					SOFSURF > −1 FN T-score ≤−2.5	92	37		
					MOST ≥ 4 FN/LS T-score ≤−2.5	Development: 80.2 Validation: 100	Development: 55.5 Validation: 67.6		
Ma et al. (2016) [33]	To compare the performance of OSTA and BFH in determining osteoporosis among postmenopausal Han Chinese women	Community-dwelling Han Chinese postmenopausal women with age range of 40–89 years (mean age: 60.71 ± 8.47 years)	1721	DXA Hologic Discovery QDR Wi BMD at LS, FN and TH	OSTA < −1 T-score at any sites <−2.5	65.28	77.15	0.782	Subjects from a single centre
					BFH-OST < −9.1 T-score at any sites <−2.5	73.58	72.66	0.797	
Lin et al. (2017) [32]	To assess the performance new screening tool to determine osteoporosis	Development phase: Community-dwelling Han Chinese males aged 50 and above (mean age: 65.42 ± 8.8) Validation phase: Hospital-dwelling Han Chinese men	Development: 1870 Validation: 574	DXA Discovery Wi, QDR series, Hologic, Waltham, MA, USA BMD at hip and LS	Development: BFH-OSTM ≤ 70 T-score < −2.5	84.96	53.49	0.763	
					Validation: OSTA ≤ −1 T-score < −2.5	50.42	82.20	0.732	
					BFH-OSTM ≤ 70 T-score < −2.5	89.92	48.57	0.795	
Satyaraddi et al. (2017) [34]	To evaluate the performance of OSTA and Male Osteoporosis Risk Estimation Score (MORES) in predicting OP among South Indian rural elderly men	Indian men aged 65 and above (mean age: 71.9 ± 5.2 years) recruited by cluster random sampling	512	DXA Hologic QDR4500 Discovery A BMD at LS and FN	OSTA ≤ 2 LS T-score ≤ −2.5	94	17	0.716	Further validation study is needed for a larger cohort of subjects
					FN T-score ≤ −2.5	99	18	0.778	
					MORES ≥ 6 LS T-score ≤ −2.5	98	15	0.855	
					FN T-score ≤ −2.5	98	13	0.760	

Abbreviation: AP, anteroposterior; AUC, area under curve; BMD, bone mineral density; BWC, body weight criteria; LS, lumbar spine; FN, femoral neck; TH, total hip; MOF, Major osteoporotic fracture; OP, osteoporosis; PF, proximal femur.

**Table 2:** Performance of OST for non-Asians.

Study	Objective	Subject Description	Number of Subjects Recruited	Methods	Cutoff	Sensitivity (%)	Specificity (%)	AUC	Remarks
Richy et al. 2004 [10]	To validate and compare the performance of OST with other osteoporosis risk indices	Postmenopausal White women (mean age: 61.5 ± 8.8 years) without Paget’s disease or advanced osteoarthritis	4035	DXA: Hologic QDR 2000 BMD at any site	OST < 2 T-score ≤ −2.5 T-score ≤ −2	86 82	40 44	0.726 0.713	Subjects were either referred or came spontaneously for osteoporosis evaluation and may differ in some ways from the general population
					SCORE > 7 T-score ≤ −2.5 T-score ≤ −2	86 78	40 46	0.708 0.700	
					ORAI > 8 T-score ≤ −2.5 T-score ≤ −2	76 73	48 51	0.670 0.668	
					OSIRIS < 1 T-score ≤ −2.5 T-score ≤ −2	64 58	69 73	0.730 0.717	
Cadarette et al. 2004 [38]	To validate the performance of osteoporosis risk indices to determine women at high risk of osteoporosis	Women (mean age: 62.4 years) with age range of 45–90 years	644	DXA BMD at FN and LS	ORAI > 8 T-score < −2.5	92.5	38.7	0.80	The study included data from women who have been selected for BMD testing
					OST chart <2 T-score < −2.5	91.5	45.7	0.82	
					OST equation < 2 T-score < −2.5	95.3	39.6	0.82	
					Body weight criterion < 70 kg	93.4	34.6	0.73	
Adler et al. 2003 [40]	To assess the performance of OST in men	American men (mean age: 64.3 ± 12.3 years) recruited from pulmonary and rheumatology clinic	181	Hologic QDR 4500 (Hologic, Inc., Bedford, MA, USA) BMD at LS, FN and TH	OST = 3 T-score ≤ −2.5	93	66	0.836	The study was not designed specifically to validate OST Small sample size
					OST= 3 T-score ≤ −2.0	74	72	0.815	
Ghazi et al. (2007) [41]	To evaluate the performance of OST in predicting men with low BMD	White men (age range: 50–85 years) from a hospital in Morocco	229	DXA Lunar Prodigy Vision machine (GE) BMD at TH and LS	OST = 2 TH T-score ≤ −2.5	87.5	58.2	0.787	
					OST = 2 LS T-score ≤ −2.5	63.6	59.5	0.660	
					OST = 2 T-score ≤ −2.5 at any site	64	60.3	0.667	
Lynn et al. (2008) [43]	To evaluate the use of OST, Male Osteoporosis Screening Tool (MOST) and Quantitative Ultrasound Index (QUI) and body weight as osteoporosis screening tools	Caucasian and Hong Kong Chinese men, aged ≥ 65 years and community-dwelling from Osteoporotic Fractures in Men (MrOS) Study	4658 Caucasian men 1914 Hong Kong Chinese men	DXA Hologic QDR 4500 W (Hologic Inc.) BMD at LS and PF	**Caucasian:**				
					OST ≤1 T-score at any site ≤ −2.5	79.3	48.5	0.714	
					OST ≤2 T-score at any site ≤ −2.5	87.6	36.1		
					MOST ≤26 T-score at any site ≤ −2.5	88.5	50	0.799	
					MOST ≤27 T-score at any site ≤ −2.5	94.7	37.8		
					**Chinese:**				
					OST ≤−2 T-score at any site ≤ −2.5	81.8	56.2	0.759	
					OST ≤−1 T-score at any site ≤ −2.5	91.9	36.4		
					MOST ≤21 T-score at any site ≤ −2.5	86.8	59.3	0.831	
					MOST ≤22 T-score at any site ≤ −2.5	94.2	42.3		
Gourlay et al. (2005) [39]	To compare the performance of three osteoporosis risk indices in two different age groups.	Postmenopausal women aged 45–96 years	4035	DXA: Hologic QDR 1000, 2000 and 4500 (Hologic Inc., Waltham, MA, USA) BMD at FN	OST ≤ 1 Ages 45−64 years	89.2	45	0.768	Subjects from a single centre
					OST ≤ −1 Ages ≥ 65 years	84.6	47.5	0.762	
					ORAI ≥ 8 Ages 45−64 years	88.5	46.2	0.750	
					ORAI ≥ 13 Ages ≥ 65 years	89.2	44.7	0.747	
					SCORE ≥ 7 Ages 45−64 years	88.5	39.8	0.757	
					SCORE ≥ 11 Ages ≥ 65 years	88.8	42.3	0.745	
Sinnott et al. (2006) [42]	To assess the performance of QUS, OST, WBC and BMI to predict low BMD in African American	African American men (age: 35 and above) recruited from clinics	128	DXA: GE Lunar (General Electric, Madison, WI, USA) BMD at LS and non-dominant hip QUS Achilles Plus System (Lunar, Madison, WI, USA)	QUS ≤ −1 T-score ≤ −2.0	83	71	0.80	Small sample size
					OST < 4 T-score ≤ −2.0	83	57	0.83	
					WBC < 85 kg	74	50	0.70	
					BMI ≥ 30	83	43	0.70	
Machado et al. (2009) [44]	To compare three different OP risk indices at different cutoffs in determining individuals who are at risk of OP	Portuguese men age 50 and above (mean age: 63.77 ± 8.22 years)	202	DXA: Hologic QDR4500/c BMD at LS and PF	OST < 1 OST < 2 OST < 3 OST < 4	47.1 61.8 75.5 85.3	72.6 63.7 50.0 32.7	0.598 0.627 0.632 0.590	
					OSTA < 1 OSTA < 2 OSTA < 3 OSTA < 4	38.2 55.9 73.5 76.5	82.1 67.9 58.3 42.9	0.602 0.619 0.659 0.597	
					BWC < 65 kg BWC < 70 kg BWC < 75 kg BWC < 80 kg	26.5 47.1 73.5 82.4	89.3 77.4 61.3 35.7	0.579 0.622 0.674 0.590	
Richards et al. (2014) [57]	To determine the performance of OST in predicting osteoporosis in males.	Male US veterans above 50 years recruited from VA Medical Centers	518	DXA: Hologic (Bedford, MA, USA) BMD at TH, LS or DF	OST ≤ 6 T-score ≤ −2.5	82.6	33.6	0.67	DXA machines from differed manufacturers were used and the results were not standardized.
Crandall et al. [54] [47]	To compare the performance of USPSTF (FRAX) with OST and SCORE to predict osteoporosis	Women aged 50–64 years who participated Women’s Health Initiative Observational Study and Clinical Trials at three of the 40 clinical centres	5165	DXA Hologic QDR2000 or QDR4500 (Bedford, MA, USA) BMD at hip or LS	USPSTF (FRAX ≥ 9.3%) FN T-score ≤ −2.5	34.1	85.8	0.60	
					OST <2 FN T-score ≤ −2.5	79.8	66.3	0.73	
					SCORE >7 FN T-score ≤ −2.5	74	70.8	0.72	
Geusens et al (2002) [51]	To compare the performance of 4 osteoporosis risk indices in determining postmenopausal women with low BMD	Women (45 years and above) from US clinic, Rotterdam Study (55 years and above), women screened for a clinical trial (55 to 81 years old) and women from the general clinic (50 to 80 years)	1102 women from US clinic 3374 women from Rotterdam Study 23,833 women screened for a clinical trial 4204 women from the general clinic	DXA Hologic (Waltham, MA, USA); Norland (Fort Atkinson, WI, USA); and Lunar (Madison, WI, USA) BMD at FN or LS	OST <2 T-score ≤−2.5	88	52	Value not mentioned	Large sample size Selection bias may occur
					ORAI >8 T-score ≤−2.5	90	52		
					SCORE >7 T-score ≤−2.5	89	58		
					SOFSURF >−1 T-score ≤−2.5	92	37		
Wallace et al. (2004) [48]	To compare the performance of five osteoporosis risk indices in determining postmenopausal African-American women with low BMD	Women (mean age: 59.4 ± 12.5 years) from an osteoporosis study	174	DXA Hologic QDR 2000 BMD at FN	ABONE ≥ 2 T-score ≤ −2.5	73.0	59.6	Value not mentioned	Small sample size
					ORAI ≥ 9 T-score ≤ −2.5	65.6	78.9		
					OST < 2 T-score ≤ −2.5	75.4	75.0		
					SCORE ≥ 6 T-score ≤ −2.5	83.6	53.9		
					Weight Criterion < 70 kg T-score ≤ −2.5	68.9	69.2		
Zimering et al. (2007) [55]	To compare a novel osteoporosis screening tool with OST in predicting low BMD	Development phase: Caucasian men (mean age: 68.4 ± 10.2 years) Validation phase: Caucasian men (mean age: 68.4 ± 10.2 years) African American men (mean age: 60.9 ± 13 years)	Development: 639 Caucasian men Validation: 197 Caucasian men 134 African American	DXA Hologic QDR 4500 SL machine (Waltham, MA, USA) BMD at FN, TH and LS	**Caucasian** M_score_ (cutoff = 9) FN T-score ≤ −2.5	88	57	0.84	M_score_ is the first validated risk assessment tool developed in men
					OST (cutoff= 4) FN T-score ≤ −2.5	85	51	0.81	
					M score age-weight (cutoff = 9) FN T-score ≤ −2.5	85	58	0.81	
					**African American** M_score_ = 9 FN T-score ≤ −2.5	NT	NT	NT	
					OST (cutoff = 4) FN T-score ≤ −2.5	100	72	0.99	
					M_score_ age-weight (cutoff = 9) FN T-score ≤ −2.5	100	73	0.99	
Jiang et al. (2016) [52]	To compare the performance of screening tools with BMI alone in identifying early postmenopausal women with OP	Postmenopausal women (mean age: 57 ± 4.2 years)	445	DXA	BMI < 28	95	38	0.73	Small sample size Low statistical power of detecting the difference in AUCs
					OST < 2 T-score ≤ −2.5	79	56	0.73	
					ORAI ≥ 9 T-score ≤ −2.5	74	60	0.69	
					SCORE ≥ 6 T-score ≤ −2.5	92	34	0.75	
					USPSTF ≥ 9.3%	24	83	0.62	
					RF ≥ 1 risk factors	66	62	0.64	
Pecina et al. (2016) [53]	To compare the effectiveness of risk tools to predict OP in women aged 50–64	Retrospective data of women (mean age: 56.6 ± 3.4) who underwent DXA scan in a clinic	290	DXA BMD at hip/LS	USPSTF FRAX ≥ 9.3%	36	73	0.55	
					SCORE ≥ 6	74	42	0.58	
					OST < 2	56	69	0.63	
					ORAI ≥ 9	52	67	0.60	
Hawker et al. (2012) [56]	To develop a screening tool to guide bone density testing in healthy mid-life women	Healthy women (age range 40–60) receiving their first BMD in an urban teaching hospital	944	DXA Lunar Prodigy (GE Healthcare, Madison WI, USA) BMD at FN, TH and LS	New tool T-score ≤ −2.0	93	36	0.75	Only Caucasian population is involved
					OST ≤1 T-score ≤ −2.0	47	Value not mentioned	0.69	
Cook et al. (2005) [49]	To assess the performance of various osteoporosis screening tools and quantitative ultrasound in relation to DXA scan	Postmenopausal women (age range: 29–87 years) recruited from DXA scanning clinics	208	DXA Hologic QDR 4500 C (Hologic Inc., Bedford, MA, USA) BMD at LS and PF	OST < −1 T-score ≤ −2.5	0.52	0.82	0.716	
					SCORE T-score ≤ −2.5	0.5	0.83	0.720	
					ORAI T-score ≤ −2.5	0.43	0.86	0.664	
					QUS BUA calcaneus T-score ≤ −2.5	0.56	0.92	0.766	
					VOS calcaneus T-score ≤ −2.5	0.61	0.72	0.723	
Perez-Castrillon et al. (2007) [46]	To identify if the combination of OST and calcaneal DXA improves the diagnosis of OP	Males with a mean age of 47 ± 13 years and females with mean age of 66 ± 8 years recruited from two university hospitals	67 males 94 females	DXA: Pixi-Lunar, DPXL Lunar (Madison, WI, USA) and Hologic QDR-4500; Hologic Inc. (Bedford, MD, USA) BMD at right calcaneal and hip	Men OST≤3 T-score < −2.5	39	86	0.623	Small sample size
					Women OST ≤ 2 T-score < −2.5	94	59	0.762	
Richards et al. (2009) [47]	To evaluate the performance of OST in predicting low BMD in male patients with rheumatoid arthritis	Males (mean age: 65.4 ± 10.5 years) recruited from a multicenter registry of rheumatoid arthritis	795	DXA Hologic Inc. (Bedford, MA, USA) BMD at Femur and LS	OST ≤ 4	64	54	Not mentioned	Low lean body mass in RA could limit the utility of the OST in this population

Abbreviation: AP, anteroposterior; AUC, area under curve; BMD, bone mineral density; LS, lumbar spine; FN, femoral neck; TH, total hip; NT, not tested; OP, osteoporosis; PF, proximal femur; QUS, quantitative ultrasound; RF, Risk Factor-Based Approach; USPSTF, the U.S. Preventive Services Task Force; WBC, Weight-based Criterion.

**Table 3:** Performance of OST to predict fracture risk.

Study	Objective	Subject Description	Number of Subjects Recruited	Methods	Cutoff	Sensitivity (%)	Specificity (%)	AUC	Remarks
Yang et al. (2013) [53]	To validate the performance of OSTA in determining vertebral fracture among postmenopausal women in China	Postmenopausal women (average age: 62 years) recruited from OP clinic in Beijing, China	1201	DXA Hologic, Inc. (Bedford, MA, USA) BMD at LS, FN and TH	OSTA < −1 and fracture	81.7	66	0.812	All subjects are recruited from one single OP centre
Crandall et al. (2014) [54]	To compare the performance of USPSTFS, OST and SCORE in predicting fracture risk among postmenopausal women	Postmenopausal women aged 50–64 years who participated in Women’s Health Initiative Observational Study and Clinical Trials	62,492	DXA Hologic QDR2000 or QDR4500 (Bedford, MA, USA) BMD at hip or LS	USPSTF(FRAX) ≥9.3%	25.8	83.3	0.56	
					SCORE > 7	38.6	65.8	0.53	
					OST < 2	39.8	60.7	0.52	
Lin et al. (2016) [55]	To validate the use of three tools in predicting new osteoporotic fractures in older Chinese men	Han Chinese men aged 50 and above	496	DXA Discovery Wi, QDR, Hologic (Waltham, MA, USA) BMD at FN, LS and TH	TH T-score < −1.4	67.57	65.45	0.711	Subjects from a single centre Two different groups of population were involved OSTA less effective in predicting risk
					FN T-score < −2.5	42.34	89.87	0.706	
					LS T-score < −1.6	52.25	77.14	0.706	
					FRAX > 2.9	81.98	62.08	0.738	
					OSTA < −1.2	53.15	76.88	0.661	
Liu et al. (2017) [56]	To evaluate the performance of Singh score and OSTA in predicting hip fracture in patients with type 2 diabetes mellitus	Postmenopausal women with 87 of them (age range: 56–86 years) had a hip fracture	261	DXA Discovery W, Hologic, Inc. (Bedford, MA, USA) BMD at hip and LS Retrospective Singh score: Standard digital anteroposterior radiographs	LS T-score < −1.85	60.9	77	0.747	Small sample size
					TH T-score < −2.45	52.9	71.8	0.699	
					FN T-score <−2.05	74.7	47.1	0.659	
					Femoral trochanter T-score <−2.25	50.6	69.5	0.631	
					OSTA < −2.5	44.8	73.8	0.534	
					Singh index < 2.5 OSTA and Singh	42.5 Value not mentioned	88.2 Value not mentioned	0.636 0.795	

AUC, area under curve; BMD, bone mineral density; LS, lumbar spine; FN, femoral neck; TH, total hip; OP, osteoporosis; USPSTF, the U.S. Preventive Services Task Force.
